# When might we consider using the newer β-lactam agents as empirical therapy against presumed carbapenem-resistant gram-negative infections?

**DOI:** 10.1093/jacamr/dlag174

**Published:** 2026-08-17

**Authors:** Stelios F Assimakopoulos, Sofia Ioannou, Jesús Rodriguez-Bano, Markos Marangos, George L Daikos

**Affiliations:** Department of Internal Medicine and Division of Infectious Diseases, University of Patras School of Medicine, Patras, Greece; Department of Medicine, Hygeia Hospital, Athens, Greece; Unidad Clínica de Enfermedades Infecciosas y Microbiología, Hospital Universitario Virgen Macarena, Departamento de Medicina, Universidad de Sevilla, Instituto de Biomedicina de Sevilla (IBiS)/CSIC, Seville, Spain; CIBERINFEC, Instituto de Salud Carlos III, Madrid, Spain; Department of Internal Medicine and Division of Infectious Diseases, University of Patras School of Medicine, Patras, Greece; Department of Medicine, Hygeia Hospital, Athens, Greece; National and Kapodistrian University of Athens, Athens, Greece

## Abstract

**Background:**

Carbapenem-resistant Gram-negative (CRGN) pathogens—including *Klebsiella pneumoniae, Pseudomonas aeruginosa* and *Acinetobacter baumannii*—are a major global health threat. Newer β-lactam agents including novel β-lactam/β-lactamase inhibitor combinations and other recently introduced β-lactam agents have expanded treatment options. However, although current guidelines focus on their use as targeted therapy, their role in empirical treatment of suspected bacterial sepsis remains unclear.

**Methods:**

In this review we evaluate epidemiological studies, randomized trials, comparative observational studies, international guidelines, microbiological surveillance data on CRGN infections and clinical factors supporting consideration of empirical use of newer β-lactam agents in patients with sepsis at risk for CRGN infection.

**Results:**

Carbapenem resistance epidemiology varies substantially across regions and strongly affects the likelihood of CRGN infection. Key risk factors include prior CRGN colonization or infection, recent broad-spectrum antibiotic exposure, prolonged hospitalization and healthcare contact in high-prevalence settings. In sepsis, delayed effective antimicrobial therapy—particularly in septic shock or severe immunosuppression—is associated with poorer outcomes. However, indiscriminate empirical use of newer β-lactam agents may promote resistance and compromise stewardship efforts. Direct evidence supporting empirical use of these agents remains limited and is based largely on observational data and extrapolation from targeted-therapy studies. Empirical use of newer agents may be appropriate for carefully selected patients with a high probability of CRGN infection and clinical scenarios where delayed active therapy could have serious consequences.

**Conclusion:**

A risk-stratified, stewardship-integrated approach incorporating local epidemiology, patient-level risk factors, illness severity and rapid diagnostics may optimize timely effective therapy while preserving the long-term utility of these agents.

## Introduction

Carbapenem-resistant Gram-negative (CRGN) bacteria—particularly *Klebsiella pneumoniae*, *Pseudomonas aeruginosa* and *Acinetobacter baumannii*—are classified by the World Health Organization as critical-priority pathogens.^1^ Over the past decade, the prevalence of CRGN organisms has increased substantially in Europe and worldwide, leading to severe infections associated with excess morbidity, mortality and healthcare costs.^2^ The accelerating global burden of antimicrobial resistance (AMR), together with the limited therapeutic options available for CRGN pathogens, has stimulated the development of several novel β-lactam agents including new β-lactam/β-lactamase inhibitor (BL/BLI) combinations and other targeted antibiotics.^3^

Several of these agents have recently entered clinical practice. These newer agents include the cephalosporin–β-lactamase inhibitor combinations ceftolozane/tazobactam and ceftazidime/avibactam; the carbapenem–β-lactamase inhibitor combinations imipenem/cilastatin/relebactam and meropenem/vaborbactam; the monobactam–β-lactamase inhibitor combination aztreonam/avibactam; the dual β-lactamase inhibitor combination sulbactam/durlobactam; and the siderophore cephalosporin cefiderocol. The literature addressing their microbiological spectrum, pharmacokinetics, clinical efficacy, safety and stewardship issues related to the use of newer β-lactam agents in critically ill patients has been comprehensively reviewed recently.^4,5^ Current guidance from the Infectious Diseases Society of America (IDSA) and guidelines from the European Society of Clinical Microbiology and Infectious Diseases (ESCMID) primarily addresses the role of these agents in targeted therapy for infections caused by multidrug-resistant Gram-negative organisms.^6,7^ In contrast, their potential role in empirical therapy remains poorly defined, as direct evidence supporting empirical use is scarce and most available data derive from studies evaluating targeted therapy after microbiological documentation.

The Surviving Sepsis Campaign recommends prompt administration of antimicrobial therapy—ideally within 1 h—for patients with suspected infection and sepsis or septic shock, although the certainty of supporting evidence remains low.^8^ Early effective antimicrobial therapy is widely considered a cornerstone of sepsis management. However, the magnitude of its survival benefit remains debated, with reported effect sizes ranging from minimal to substantial.^9–12^ Moreover, a substantial proportion of patients treated empirically for presumed sepsis—estimated at up to 60%—are ultimately found not to have a culture-positive infection.^13^ Consequently, empirical treatment strategies frequently expose patients to broad-spectrum antibiotics unnecessarily, potentially contributing to toxicity, ecological disruption and the selection of further AMR.^12^

The availability of newer agents with reliable activity against CRGN pathogens therefore creates a therapeutic dilemma. On one hand, delayed administration of effective therapy in patients with severe infections caused by resistant organisms may worsen clinical outcomes.^14–17^ On the other hand, indiscriminate empirical use of newer β-lactam agents may accelerate resistance development, increase ecological pressure and compromise the long-term effectiveness of these agents. Determining when the probability of CRGN infection justifies empirical use of these valuable agents therefore represents an important clinical challenge.

In the setting of limited and largely indirect evidence supporting the empirical use of newer β-lactam agents, our aim is to address a key practical question: under which clinical and epidemiological circumstances may these agents be considered for empirical therapy in patients with sepsis? In this narrative review, we summarize the available evidence and integrate epidemiological, microbiological, host-related and stewardship considerations into a pragmatic decision-support framework that may assist clinicians when considering empirical use of newer β-lactam agents in carefully selected patients with suspected CRGN sepsis.

## Methods

This narrative review was developed through an expert-based review of the available evidence addressing sepsis, epidemiology and treatment strategies for CRGN infections, with a particular focus on the potential role of newer β-lactam agents as empirical therapy. Relevant studies and guidance documents published between 2000 and 2026 were identified through structured searches of PubMed and manual screening of reference lists. Search terms included combinations of: ‘empirical therapy’, ‘carbapenem-resistant Gram-negative bacteria’, ‘β-lactam/β-lactamase inhibitors’, ‘novel β-lactam’, ‘sepsis and septic shock’, ‘antimicrobial stewardship’, ‘carbapenem-resistant *Pseudomonas*’ (CRPA), ‘carbapenem-resistant Enterobacterales’ (CRE) and ‘carbapenem-resistant *A. baumannii*’ (CRAB).

Priority was given to international guidelines, randomized controlled trials, comparative observational studies, epidemiological surveillance data and clinically relevant evidence regarding newer antimicrobial agents. Given the limited direct evidence supporting the empirical use of newer β-lactam agents against CRGN infections, the findings presented should be interpreted as a pragmatic, risk-based discussion rather than formal evidence-graded recommendations.

### Epidemiology of carbapenem-resistant infections: a driver of empirical use of new β-lactam agents

Local epidemiology is a key determinant of empirical antibiotic selection. The prevalence of resistant pathogens, the distribution of causative organisms and AMR profiles vary substantially between hospitals, regions and continents. In large international ICU cohorts, Gram-negative pathogens predominated in several high-AMR regions, including Eastern Europe, Africa and parts of Asia and the Middle East.^18^ Several studies have shown that among Gram-negative pathogens, carbapenem resistant strains are associated with the highest attributable mortality, particularly in bloodstream infections and ventilator-associated pneumonia.^19,20^

The global prevalence of carbapenem resistance continues to increase with marked geographic heterogeneity.^2^ In Europe, carbapenem resistance follows a pronounced gradient, increasing from north to south and from west to east. According to the latest report from the European Antimicrobial Resistance Surveillance Network (EARS-Net 2025), mean carbapenem resistance rates for *K. pneumoniae*, *P. aeruginosa* and *A. baumannii* were 11.3% (range 0.0%–67%), 15.9% (range 1.5%–53.4%) and 31.6% (range 0.0%–94.1%), respectively.^21^ Such variability underscores that empirical use of agents targeting CRGN pathogens cannot be generalized and must be guided by local epidemiology and institutional surveillance data.

When selecting empirical antimicrobial therapy, it is also essential to consider the underlying mechanisms of carbapenem resistance, as these directly influence the activity of available agents. In Enterobacterales, carbapenem resistance is primarily mediated by carbapenemases, most commonly Ambler class A (KPC), class D (OXA-48-like) and class B metallo-β-lactamases (MBLs; e.g. VIM, IMP, NDM). Their geographic distribution varies substantially: MBLs predominate in Asia-Pacific (59.4%) and parts of Africa and the Middle East (49%), OXA-48-like enzymes are common in Europe (30%) and KPC predominate in Latin America (51.9%) and North America (53.4%).^22^ Importantly, the epidemiology of carbapenemases is continuously evolving, with an increasing dissemination of MBL-producing Enterobacterales reported in several European countries, underscoring the importance of continuous local surveillance when selecting empirical therapy.^23^ Local institutional epidemiology may further vary because of clonal dissemination and outbreaks. In *P. aeruginosa*, carbapenem resistance often results from multiple mechanisms including porin loss, efflux pump overexpression, AmpC overproduction and occasionally acquired carbapenemases (mostly VIM, NDM, KPC, GES).^6^ Similarly, resistance in *A. baumannii* reflects multiple coexisting mechanisms, including chromosomal class C (ADC) and class D (OXA-51 family) β-lactamases together with a variety of acquired carbapenemases (OXA-23, OXA-24, OXA-58, OXA-134, NDM, VIM, KPC) depending on the geographic region.^24^

**Figure 1.**
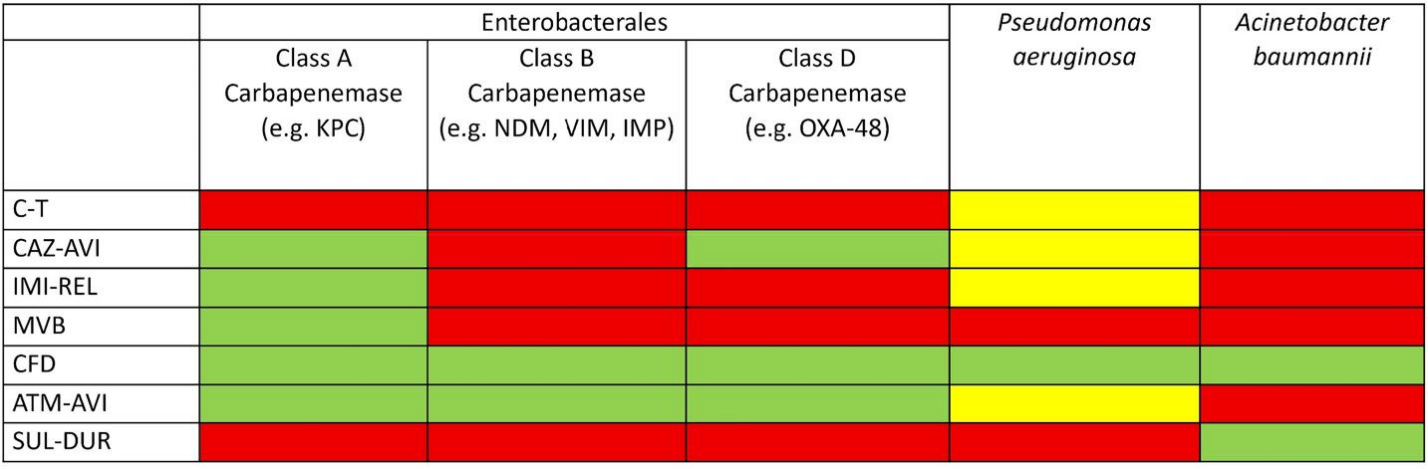
Expected *in vitro* activity of newer β-lactam agents against carbapenem-resistant Gram-negative pathogens according to predominant carbapenem resistance mechanisms. This figure summarizes the expected *in vitro* activity of the listed agents according to the predominant carbapenem resistance mechanisms. Activity may vary depending on local epidemiology and resistance mechanisms. In particular, reduced susceptibility to cefiderocol has been reported among some NDM-producing isolates. C–T, ceftolozane/tazobactam; CAZ–AVI, ceftazidime/avibactam; IMI–REL, imipenem/relebactam; MVB, meropenem/vaborbactam; CFD, cefiderocol; ATM–AVI, aztreonam/avibactam; SUL-DUR, sulbactam/durlobactam. Green: expected to be active; Red: expected to be non-active; Yellow: expected to have variable activity depending on underlying mechanism of resistance (exceptions from these colour designations might exist).

These diverse resistance mechanisms have important therapeutic implications, as not all newer β-lactam agents retain activity across carbapenemase classes (Figure 1). Even cefiderocol, which has the broadest *in vitro* spectrum, demonstrates resistance rates exceeding 35% among NDM-producing Enterobacterales, *Pseudomonas* and *Acinetobacter* isolates.^25^ Furthermore, resistance among KPC-producing Enterobacterales to newer BL/BLI combinations is increasingly reported.^26^ Empirical administration of a single novel agent without knowledge of the local molecular epidemiology may therefore result in therapeutic mismatch.

Beyond regional epidemiology, patient-level factors strongly influence the probability of CRGN infection. Prior colonization or infection with carbapenem-resistant organisms, recent exposure to carbapenems or other broad-spectrum antibiotics, prolonged hospitalization, ICU stay and healthcare exposure in high-prevalence regions are consistently identified as major risk factors.^27,28^ Pragmatically, the probability of CRGN infection is likely highest in patients with recent colonization or infection by these organisms, prolonged ICU exposure, recent broad-spectrum antibiotic use and hospitalization in units with high endemicity or ongoing outbreaks. Approximately 20% of patients colonized with CRE subsequently develop infection with the same organism.^29–31^ Notably, the risk of subsequent CRE infection is highest during the early period following colonization, highlighting the importance of timing in risk stratification.^30,32^ The site of colonization may be related to the location of infection; for example, the risk for pneumonia in patients with rectal carriage of CRE is probably lower compared with intra-abdominal infection. Also, the risk appears to be higher in severely immunocompromised populations, reaching 25.8% in recipients of autologous stem cell transplantation, 39.2% in those undergoing allogeneic transplantation and 70% in liver transplant recipients.^33,34^ Although most evidence derives from CRE-colonized patients, emerging data indicate that colonization with CRAB or CRPA is also associated with a substantially increased risk of subsequent infection, particularly among critically ill, mechanically ventilated and immunocompromised patients.^35,36^ Although prior colonization with CRGN is consistently one of the strongest predictors of subsequent infection, a substantial proportion of colonized patients do not develop infection with the colonizing strain. These observations highlight the need for improved predictive approaches that integrate colonization status with additional host, clinical and epidemiological factors. Such an approach could help to identify patients with a sufficiently high probability of CRGN infection to justify early empirical therapy directed against CRGN pathogens while minimizing unnecessary use of newer β-lactam agents and reserving them for patients that would benefit most.

### The impact of empirical antimicrobial treatment in sepsis

A substantial proportion of patients with sepsis receive inappropriate initial antimicrobial therapy, particularly in healthcare settings where AMR is prevalent.^37–41^ Large cohort analyses estimate that inappropriate empirical therapy occurs in approximately 20%–40% of septic patients, with even higher rates in infections caused by multidrug resistant organisms.^37–39,42,43^

Inappropriate initial therapy has repeatedly been associated with worse clinical outcomes including increased mortality, prolonged hospital stays, delayed clinical recovery and higher healthcare burden.^39–41,43–45^ This association has been reported across multiple infection syndromes, including bloodstream infections, hospital-acquired pneumonia (HAP) and intra-abdominal infections. Although residual confounding cannot be excluded, the consistency of these findings supports the concept that early effective antimicrobial therapy is an important contributor to outcome in severe infections.

The relationship between time to antibiotic administration and mortality, however, remains more controversial. Early observational studies suggested that delays in antimicrobial administration after recognition of sepsis were associated with progressively increasing mortality, leading to strong recommendations for early broad-spectrum therapy.^9,46^ A meta-analysis reported reduced mortality when appropriate antimicrobial therapy was administered within the first hour of sepsis recognition.^47^ In contrast, other systematic reviews have not demonstrated a clear linear relationship between each hour of delay and increased mortality.^10^ These discrepant findings likely reflect methodological limitations inherent to observational studies, including variability in definitions of sepsis onset and incomplete adjustment for illness severity and other confounding factors. These considerations highlight the need to balance timely active therapy with avoidance of unnecessary broad-spectrum antimicrobial exposure, particularly in settings where the probability of resistant infection is uncertain.

Importantly, the clinical impact of delays in effective therapy appears to be heterogeneous across patient populations. Several studies suggest that the benefit of early antimicrobial administration is substantially greater in patients with septic shock than in those with less severe forms of sepsis.^11,48^ In one large cohort analysis, each hour of delay in antibiotic administration was associated with a modest increase in absolute mortality among patients with sepsis without shock, but a substantially greater increase among those with septic shock.^11^ Specifically, the estimated increase in absolute mortality per hour of delay was 0.3% for sepsis, 0.4% for severe sepsis and 1.8% for septic shock.

Host-related factors further modify the consequences of delayed or inappropriate therapy. Patients with severe immunosuppression—such as those with neutropenia, hematologic malignancies, or recipients of hematopoietic stem cell transplantation—are particularly vulnerable to rapid clinical deterioration when effective antimicrobial therapy is delayed.^49^ In these populations, early administration of appropriate therapy is considered a cornerstone of sepsis management and may have a greater impact on survival than in immunocompetent hosts.^50^

Taken together, these observations indicate that both the timeliness and appropriateness of empirical antimicrobial therapy influence outcomes in sepsis, although the magnitude of benefit varies across clinical contexts.

### Empirical antimicrobial therapy for suspected carbapenem-resistant infections: the potential role of newer β-lactam agents

The decision to incorporate the newer β-lactam agents into empirical regimens for patients with presumed sepsis should not rely solely on the theoretical benefit of early effective therapy. Instead, it requires careful integration of several elements: local epidemiology, patient-specific risk factors for CRGN pathogens, severity of illness, host vulnerability, and predicted mortality risk. The objective is to maximize the likelihood of appropriate initial therapy while preserving these agents as critical antimicrobial resources.

Novel BL/BLI combinations and other newer agents have substantially improved outcomes in confirmed infections caused by CRE and other CRGN pathogens. Current guidance from the IDSA and the ESCMID guidelines supports their preferential use as targeted therapy for susceptible CRGN infections.^6,7^ Importantly, these recommendations are largely based on pathogen-specific randomized controlled trials that included relatively small numbers of patients, as well as observational studies consistently demonstrating improved survival compared with historical standard-of-care regimens.^51–68^ Notably, direct evidence supporting empirical use of these agents remains limited.^14–17,69,70^

The central question, however, is not whether these agents are effective—accumulating evidence indicates that they are in appropriately selected patients—but when their empirical use is justified. One of the most consistent findings across epidemiological studies is that prior colonization or infection with CRGN represents one of the stronger predictors of subsequent infection. Patients colonized with CRGN pathogens who develop sepsis may therefore have a substantial probability that the same organism is responsible for the infection. In contrast, in patients without risk factors for CRGN pathogens and in low-prevalence settings, the probability of CRGN infection remains low. Consequently, empirical use of newer β-lactam agents should be reserved for clinical scenarios in which the pre-test probability of CRGN infection is sufficiently high.

An additional complexity often underappreciated in discussions of empirical therapy is the heterogeneity of carbapenem resistance mechanisms. Different β-lactam agents exhibit variable activity depending on the underlying carbapenemase (Figure 1). For example, meropenem/vaborbactam and ceftazidime/avibactam demonstrate excellent activity against KPC-producing organisms but lack activity against MBLs. In regions where MBLs are prevalent, therapeutic strategies may instead require aztreonam/avibactam or combinations such as ceftazidime/avibactam plus aztreonam.^4,6,23,71^ Empirical selection of a novel BL/BLI agent without awareness of the local molecular epidemiology therefore risks therapeutic mismatch despite the use of advanced antibiotics. The availability of rapid molecular diagnostics may help reduce this uncertainty by enabling early mechanism-directed escalation or de-escalation.^72^

Host factors also play an important role in determining the acceptable threshold for empirical escalation. Patients with severe immunosuppression—including those with neutropenia, hematologic malignancies, or recipients of hematopoietic stem cell transplantation—are particularly vulnerable to rapid clinical deterioration if effective antimicrobial therapy is delayed. In such populations, empirical use of newer β-lactam agents may be justified when the probability of CRGN infection is high, as traditional salvage options such as polymyxins or aminoglycosides without β-lactams are often inadequate or poorly tolerated in profoundly immunocompromised hosts.^49,50^

For suspected CRPA infection, empirical treatment decisions should also be guided by the predominant local resistance mechanisms, as these determine the activity of the available newer β-lactam agents. As mentioned previously, carbapenem resistance in *P. aeruginosa* frequently results from porin loss, AmpC hyperproduction and efflux pump overexpression, whereas acquired carbapenemases, particularly MBLs, show marked geographical variability.^6,27^ Consequently, ceftolozane/tazobactam and imipenem/relebactam remain appropriate empirical options in regions where non-carbapenemase-mediated resistance predominates, while ceftazidime/avibactam may retain activity against selected GES-producing isolates but lacks activity against MBL-producing strains.^6,54,55,66^ In contrast, cefiderocol is the newer β-lactam with the broadest and most consistent *in vitro* activity against most MBL-producing *P. aeruginosa* isolates and may therefore represent the preferred empirical option in MBL-endemic settings, although susceptibility variability and emerging resistance require prompt microbiological confirmation and early reassessment.^4,6,25^ Accordingly, empirical use of these agents should be restricted to patients with septic shock, sepsis with progressive multiorgan dysfunction, profound immunosuppression or neutropenia who also have epidemiological risk factors for CRPA, such as previous colonization or infection or hospitalization in units with a high prevalence of these organisms. In such high-risk patients, addition of a second active agent (e.g. amikacin, tobramycin or colistin) may further increase the probability of appropriate initial therapy until susceptibility results become available (Table 1).^5–7^

**Table 1.** Suggested empirical treatment for presumed CRGN infections

Patient risk category	Presumed pathogen^a^			
	KPC-E	MBL-E	CRPA	CRAB
High risk^b^	CAZ–AVI ± (AMG or Colistin)^c^ MVB ± (AMG or Colistin)^c^ IMI–REL ± (AMG or Colistin)^c^	ATM–AVI ± (AMG or Colistin)^c^ CAZ–AVI + ATM ± (AMG or Colistin)^c^	MBL-dominant settings CFD + (Amikacin or tobramycin or colistin)	Preferred Sulbactam/durlobactam + Imipenem^d^ Alternative CFD + high-dose ampicillin/sulbactam High-dose ampicillin/sulbactam (27 g/day) + (colistin or tigecycline high dose)
			Non-MBL dominant settings C-T + (Amikacin or Tobramycin or Colistin) IMI-REL + (Amikacin or Tobramycin or Colistin) CAZ–AVI + (Amikacin or Tobramycin or Colistin)	
Low-risk	(Colistin or AMG) +Tigecycline (cIAI) (Colistin or AMG) +Fosfo (BSI, HAP/VAP, cUTI)	(Colistin or AMG) +Tigecycline (cIAI) (Colistin or AMG) + Fosfo (BSI, HAP/VAP, cUTI)	(Colistin or Amikacin or Tobramycin) + Fosfo	High-dose ampicillin/sulbactam (27 g/day) + (colistin or tigecycline high dose) CFD + high-dose ampicillin/sulbactam

Abbreviations: KPC-E, *Klebsiella pneumoniae* carbapenemase–producing Enterobacterales; MBL-E, metallo-β-lactamase–producing Enterobacterales; CRPA, carbapenem-resistant *Pseudomonas aeruginosa*; CRAB, carbapenem-resistant *Acinetobacter baumannii*; CAZ–AVI, ceftazidime/avibactam; ATM–AVI, aztreonam/avibactam; CAZ–AVI + ATM, ceftazidime/avibactam plus aztreonam; IMI–REL, imipenem/relebactam; C–T, ceftolozane/tazobactam; CFD, cefiderocol; MVB, meropenem/vaborbactam; AMG, aminoglycosides; Fosfo, fosfomycin; BSI, bloodstream infection; HAP/VAP, hospital-acquired pneumonia/ventilator-associated pneumonia; cIAI, complicated intra-abdominal infection; cUTI, complicated urinary tract infection.

Newer β lactam agents: ceftazidime/avibactam; aztreonam/avibactam; imipenem/relebactam; ceftolozane/tazobactam; cefiderocol; meropenem/vaborbactam; sulbactam-durlobactam

Conventional agents: Aminoglycosides; fosfomycin; tigecycline; polymyxins; ampicillin-sulbactam

^a^According to previous colonization/infection (last 6 months) or prevalent pathogen in ward/unit.

^b^Septic shock, or rapidly progressive organ dysfunction, or neutropenia (≤500 PMNs/μL).

^c^AMG or Colistin suggested as additional coverage for other potential pathogens (CRPA, CRAB).

^d^Sulbactam/durlobactam is suggested in combination with imipenem to cover other potential pathogens. This regimen was evaluated in the ATTACK trial and is recommended in the current IDSA guidance.

A similarly individualized approach is required for presumed CRAB infection. Because most currently available BL/BLI combinations have limited or no activity against CRAB, empirical therapy should primarily rely on agents with proven anti-*Acinetobacter* activity.^6,24^ The recent ATTACK trial established sulbactam/durlobactam plus imipenem/cilastatin as the preferred treatment for severe CRAB infections, demonstrating non-inferior efficacy with substantially lower nephrotoxicity than colistin-based therapy; this strategy has subsequently been incorporated into contemporary treatment recommendations.^6,53^ Where sulbactam/durlobactam is unavailable, high-dose ampicillin/sulbactam combined with colistin or high-dose tigecycline remains a reasonable alternative, whereas cefiderocol may be considered in selected patients, recognizing the conflicting clinical evidence, mortality concerns raised in the CRAB subgroup of the CREDIBLE-CR trial and the potential for heteroresistance.^51,59,61,63–65^ Therefore, empirical use of newer anti-CRAB agents should also be reserved for patients with septic shock, sepsis with progressive multiorgan dysfunction, neutropenia or other conditions associated with a high risk of death, together with strong epidemiological evidence suggesting CRAB infection, followed by rapid microbiological confirmation and early de-escalation whenever appropriate, in accordance with antimicrobial stewardship principles.^5–7,73^

**Figure 2.**
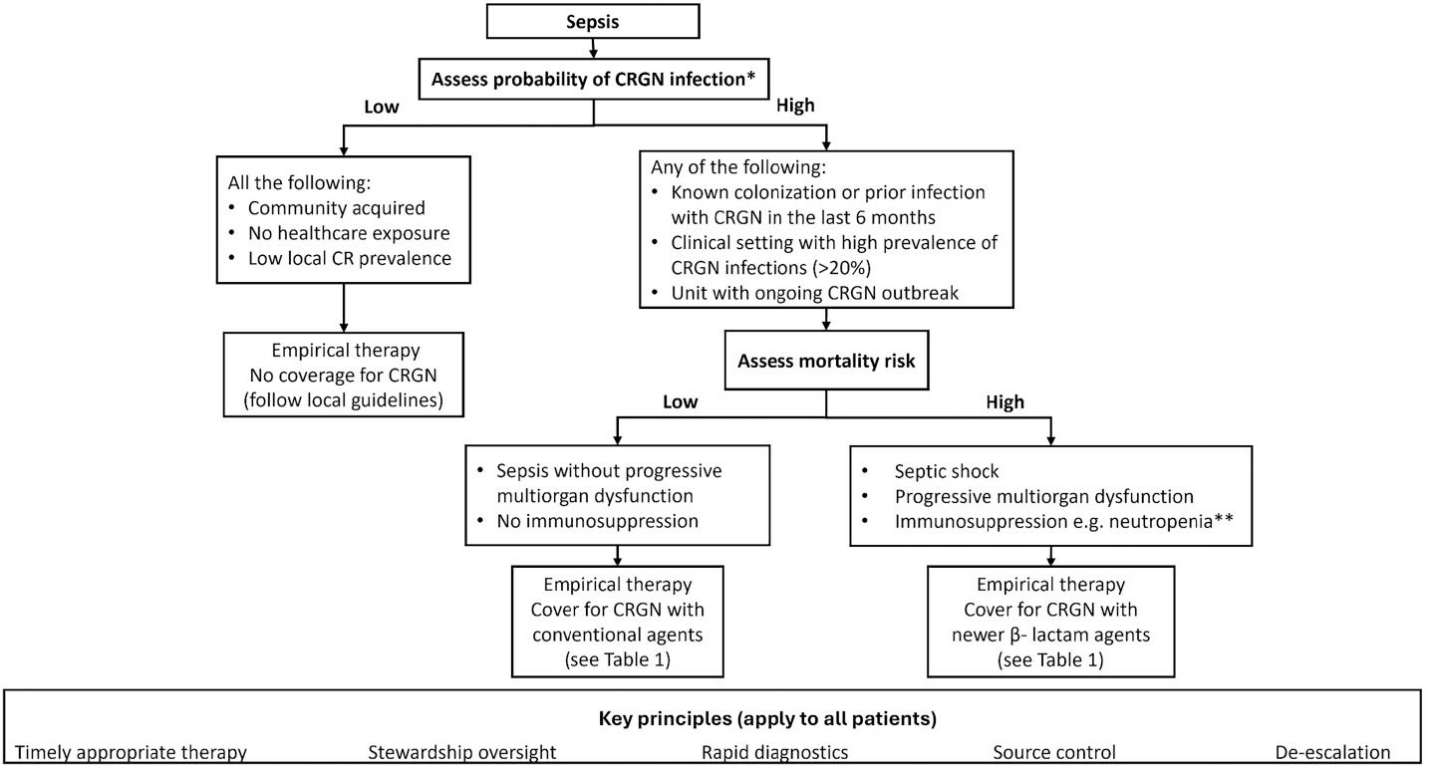
Proposed risk-stratified algorithm for empirical therapy of patients with presumed CRGN infection. This algorithm is an expert-informed decision-support framework and not a validated prediction model. In patients with high probability of CRGN infection, empirical therapy with newer β-lactam agents may be considered when any major high-mortality feature is present, including septic shock, rapidly progressive organ dysfunction, or profound immunosuppression/neutropenia. *Existing risk scores (like Giannella score) may be used to support the assessment of probability for CRGN infection.^74–78^ **Neutropenia: absolute neutrophil count ≤500 cells/μL, or ≤1000 cells/μL with an expected decline to ≤500 cells/μL within 48 h; CRGN: carbapenem resistant Gram-negative. Newer β lactam agents: ceftazidime/avibactam; aztreonam/avibactam; imipenem/relebactam; ceftolozane/tazobactam; cefiderocol; meropenem/vaborbactam; sulbactam-durlobactam. Conventional agents: Aminoglycosides; fosfomycin; tigecycline; polymyxins; ampicillin-sulbactam.

These considerations highlight that empirical treatment decisions in sepsis cannot follow a ‘one-size-fits-all’ approach. Rather, a risk-weighted strategy is required (Figure 2). In practical terms, empirical therapy with newer β-lactam agents may be reasonable when a high probability of CRGN infection, based on documented colonization or prior infection and/or hospitalization in a setting with high CRGN prevalence, coexists with any major high-mortality feature, such as septic shock, rapidly progressive organ dysfunction, or profound immunosuppression/neutropenia. The convergence of these factors markedly increases the probability that CRGN pathogens are responsible for infection and that delayed effective therapy will adversely affect outcomes. Under such circumstances, particularly septic shock in a patient with documented CRGN colonization or prior infection, empirical administration of a newer β-lactam agent may represent a rational strategy.^5^ Of note, efforts have already been made to improve identification of patients at highest risk of CRGN infection. Gianella *et al.* developed a clinical prediction model to identify CRE-colonized patients at increased risk of subsequent infection, incorporating variables such as intensive care exposure, invasive procedures, chemotherapy/radiotherapy and colonization burden. The model has subsequently undergone external validation with acceptable discriminatory performance.^74–77^ However, its applicability is limited to CRE-colonized patients and does not address other important CRGN pathogens such as CRPA or CRAB. More recently, prediction models have also been proposed for other CRGN pathogens; however, these models currently have limited external validation.^78^ Available prediction scores may refine the estimation of CRGN infection probability but cannot, by themselves, determine the need for empirical use of newer β-lactam agents, which also requires consideration of severity of illness, host vulnerability, expected mortality risk, local epidemiology and stewardship principles.

Importantly, in critically ill patients selected to receive empirical therapy with newer β-lactam agents, pharmacokinetic variability may hinder achievement of optimal antimicrobial exposure. Critically ill patients frequently exhibit profound pharmacokinetic alterations, including increased volume of distribution, augmented renal clearance or renal replacement therapy, which may substantially reduce β-lactam exposure. Current sepsis guidelines therefore support loading doses followed by prolonged or continuous β-lactam infusion, particularly in patients with septic shock, augmented renal clearance, pneumonia, or infections caused by pathogens with elevated MICs, as these strategies may improve pharmacodynamic target attainment in clinical settings characterized by substantial pharmacokinetic variability.^8^ However, this recommendation is based on the broader β-lactam literature and does not specifically differentiate the newer β-lactam agents. For these agents, available PK/PD studies and Monte Carlo simulations support optimized administration strategies, including extended infusions—commonly 3-h infusions for cefiderocol and meropenem/vaborbactam and 4 to 6-h infusions for ceftolozane/tazobactam and ceftazidime/avibactam, particularly in patients with augmented renal clearance, high MIC isolates, or difficult-to-penetrate infection sites.^4,79^ When available, therapeutic drug monitoring combined with MIC determination may further individualize dosing in patients at high risk of non-attainment PK/PD targets.

Conversely, in patients with a low mortality risk and low predicted probability of CRGN infection or in diagnostically uncertain scenarios, empirical regimens based on conventional agents and guided by local epidemiology may remain appropriate. In these situations, older antibiotics such as polymyxins, aminoglycosides, tigecycline, fosfomycin, or ampicillin–sulbactam—used in combination strategies tailored to the suspected pathogen—may provide adequate coverage while preserving newer β-lactam agents. As illustrated in Table 1, for selected patients with low-risk infection (e.g. uncomplicated urinary tract infections) who are colonized with CRGNs known to be susceptible to conventional agents, an alternative stewardship-oriented approach is to include one of these agents (e.g. an aminoglycoside or cotrimoxazole) to the empirical regimen, rather than initiating therapy with a newer β-lactam agent, while awaiting culture and susceptibility results. However, given the well-recognized nephrotoxicity and/or other adverse effects associated with several conventional agents, this approach should be accompanied by close clinical monitoring and prompt re-evaluation as soon as microbiological and susceptibility results become available. Observational data support this risk-stratified approach; for example, the CAVICOR study demonstrated that the survival advantage of ceftazidime/avibactam compared with conventional agents was most pronounced in critically ill patients with high INCREMENT scores (>7), suggesting that the benefit of novel therapies may be concentrated among those at highest risk of death.^60^

The indiscriminate use of newer β-lactam agents in empirical treatment may have detrimental ecological effects and undermine stewardship efforts. Emergence of resistance has already been documented for several of these agents, including ceftazidime/avibactam and cefiderocol, and less frequently for other BL/BLI combinations.^25,26,80^ Unrestricted empirical use may therefore accelerate resistance selection and compromise the long-term clinical effectiveness of these valuable therapeutic agents. For this reason, their deployment should be embedded within robust antimicrobial stewardship programmes.^73^ Implementation of such a risk-based empirical strategy also requires institutional preparedness. Hospitals managing patients at high risk for CRGN infections should ensure timely access to selected newer β-lactam agents through locally adapted antimicrobial stewardship policies and formulary planning.

A key stewardship principle is systematic reassessment of empirical therapy within 48–72 h or even sooner using rapid diagnostics, with prompt de-escalation once microbiological data become available. The expanding availability of rapid diagnostic technologies supports this stewardship-driven approach by enabling earlier microbiological clarification and facilitating timely modification of empirical therapy. MALDI-TOF–based workflows and molecular rapid diagnostic tests, including multiplex PCR panels, can significantly shorten the time to pathogen identification and resistance detection.^72^ Rapid diagnostic technologies encompass assays with different turnaround times and clinical applications. Some molecular assays performed directly on clinical specimens (e.g. whole blood or other biological fluids) may inform very early empirical treatment decisions by rapidly identifying pathogens or resistance determinants. In contrast, assays performed on positive blood cultures or bacterial colonies primarily facilitate earlier optimization of therapy through timely escalation or de-escalation compared with conventional microbiological methods. For example, the BioFire FilmArray Pneumonia Panel can identify respiratory pathogens and selected resistance genes directly from sputum or bronchoalveolar lavage specimens within approximately 1 h.^81^ In contrast, the BioFire Blood Culture Identification 2 Panel requires a positive blood culture and provides organism identification together with detection of selected resistance genes in approximately 1 h once the assay is performed on a positive blood culture.^82^ Similarly, the Accelerate Pheno system is performed on positive blood cultures and provides organism identification within approximately 2 h and phenotypic antimicrobial susceptibility results within approximately 7 h.^83^ T2 magnetic resonance assays can detect selected bloodstream pathogens directly from whole blood within approximately 3–5 h, without awaiting blood-culture positivity.^84^ At a later stage of the microbiological workflow, lateral-flow carbapenemase assays can rapidly identify major carbapenemase families within approximately 15 min once an isolated bacterial colony has become available.^85^ However, limitations such as incomplete resistance gene coverage, gene–phenotype discordance and variability in laboratory implementation remain important challenges.^86^ Earlier pathogen and mechanism identification enables timelier optimization of antimicrobial therapy, facilitating both targeted escalation when CRGN pathogens are confirmed and rapid de-escalation when they are excluded. Importantly, a recent meta-analysis demonstrated that integration of rapid diagnostics with stewardship interventions significantly reduces time to optimal therapy and is associated with improved survival in patients with bloodstream infections.^87^ Together, stewardship oversight and rapid diagnostics represent essential safeguards to ensure that empirical use of newer β-lactam agents remains both clinically effective and sustainable. Of note, the availability of rapid diagnostic technologies varies considerably across healthcare settings, particularly in low- and middle-income countries, where access may be limited. In such settings, the proposed risk-stratified approach in Figure 2 remains applicable but decisions for empirical treatment should rely mainly on local epidemiology, patient-level risk factors, previous colonization status and careful clinical reassessment.

Overall, newer β-lactam agents have transformed the therapeutic landscape for CRGN infections. Nevertheless, their empirical use should remain individualized and focused on carefully selected high-risk clinical scenarios. Severity of illness alone is insufficient justification for empirical anti-CRGN therapy in the absence of epidemiological or microbiological risk factors. Rather, empirical therapy targeting CRGN pathogens should be considered primarily when a high pre-test probability of CRGN infection coexists with clinical scenarios in which delayed active therapy is expected to have major consequences. A calibrated, mechanism-aware, and stewardship-integrated approach therefore represents the most rational strategy, maximizing the probability of early effective therapy for high-risk patients, while preserving the long-term effectiveness of these critical antimicrobial agents. Pending higher-quality prospective evidence, the development of an international multidisciplinary expert consensus under the auspices of scientific societies such as ESCMID and IDSA may help harmonize clinical practice and provide evidence-informed guidance for the empirical use of newer β-lactam agents.
